# 

**Published:** 1884-04

**Authors:** 


					﻿Whole Number,
CCLXIII.
APRIL, 1884.
Number
Volum
THE
BUFFALO
Medical and Surgical Journal.
EDITORSs
THOS. I.OTHROP, M.
A. R. DAVIDSON, M.
R. W. VAN PEYMA, M. D.
Published by the Medical Journal Association.
No. 3 WEST CHIPPEWA ST.
Entered at the Post-Office at Buffalo, N. Y., as Second-class Mail Matter.
				

